# Improving radiofrequency ablation outcomes in large thyroid nodules: superiority of adjustable tip needles

**DOI:** 10.3389/fendo.2026.1832999

**Published:** 2026-06-01

**Authors:** Mattia Rossi, Vittorio Voena, Barbara Lucatello, Federico Ragazzoni, Francesca Retta, Josefina Ramunni, Alberto Mormile, Maurilio Deandrea

**Affiliations:** 1 Endocrinology, Diabetology and Metabolism, AO Ordine Mauriziano di Torino, Turin, Italy; 2 School of Medicine, Department of Medical Sciences, University of Turin, Turin, Italy

**Keywords:** adjustable tip needle, benign nodule, mini-invasive treatment, radiofrequency ablation, thyroid ablation, thyroid nodule

## Abstract

**Background:**

Approximately 10–12% of thyroid nodule radiofrequency ablations (RFA) result in failure or regrowth, particularly for nodules larger than 20–30 mL. Adjustable-tip needles (ATN) have recently been introduced to allow tailored heat distribution, although current literature does not demonstrate clear advantages over fixed-tip needles (FTN). This study aimed to assess the efficacy of ATNs in nodules with baseline volume >20 mL.

**Methods:**

This retrospective, monocentric observational study included 75 patients (38 FTN, 37 ATN) who underwent RFA for benign thyroid nodules >20 mL, with at least 12 months of follow-up. All patients had confirmed cytological benignity, negative serum calcitonin, normal thyroid function, and ENT evaluation. Technical parameters (energy, duration), volume reduction ratio (VRR), and Compressive/Cosmetic scores were collected at 1, 6, and 12 months. Therapeutic success was defined as VRR ≥50%.

**Results:**

ATNs yielded greater and sustained volume reduction (VRR at 12 months: 60.5% vs 52.4%, *p* = 0.004; final volume: 14.1 vs 18.4 mL, *p* = 0.012; AUC_VRR_ *p* = 0.019; AUC_Vol_ *p* = 0.040), with a progressive time-related effect (*p*_GROUP: TIME_ = 0.001; β = 1.12). ATNs were associated with higher 12-month success rates (84% vs 55%, OR 4.18 (1.42–12.35), *p* = 0.015). Energy deposition was significantly higher. The volumetric cutoff for achieving 75% success shifted from 35 mL (FTN) to 80 mL (ATN).

**Conclusion:**

In benign thyroid nodules >20 mL, RFA with ATNs proved more effective than FTNs, thanks to a higher energy deposition. This could suggest a shift in the eligibility threshold for RFA up to 80 mL.

## 1 Introduction

Clinical and autopsy studies have shown that, partly due to overdiagnosis following suboptimal ultrasound screening, up to 60% of the general population harbor at least one thyroid nodule. However, only 7–15% of these nodules prove to be malignant, while the vast majority are benign (1). Although they carry no risk of metastatic spread, benign thyroid nodules often exhibit progressive growth and may compress surrounding structures, leading to compressive symptoms or cosmetic concerns.

Historically, the main treatment option — after a brief period of interest in radioiodine (RAI) therapy — has been surgery. While effective, thyroidectomy is not without risks, including hypothyroidism, hypoparathyroidism, and recurrent laryngeal nerve injury, as well as visible scarring, despite recent advances in minimally invasive techniques (2, 3).

Over the past two decades, ultrasound-guided radiofrequency thermal ablation (RFA) has emerged as a minimally invasive option for symptomatic benign nodules. RFA has been shown to be both effective in reducing nodule size and symptoms, and safe, with complication rates significantly lower than surgery. It is now recommended as a first-line alternative for symptomatic benign thyroid nodules by several international guidelines (4–7).

However, some limitations remain. Variables such as large baseline volume, operator experience, energy delivery, and proximity to critical structures can reduce treatment completeness, resulting in suboptimal volume reduction or regrowth over time (8–10). In response, recent innovations have focused on technical modifications to overcome these challenges. One such innovation is the adjustable-tip electrode (ATN), which allows intra-procedural modulation of the active tip length (from 5 to 30 mm), unlike the standard fixed-tip needle (FTN), typically 10 mm. This variability may reduce the need for repositioning, shorten procedure times, enhance energy distribution, lower impedance rises and carbonization risk, and potentially simplify the learning curve for novice operators.

The ATN was originally designed for hepatic tumors, and a large body of literature exists on that specific field of application, both regarding animal ex-vivo and *in-vivo* livers (11), and clinical employment on human patients (12–14). However, very few studies exist describing this approach, when considering thyroid nodules ablation (15, 16), even if several experiences have been rumored among experts.

While preliminary studies suggest procedural advantages of ATNs, current evidence is limited and largely based on short-term follow-up, with no significant differences in volume reduction ratio (VRR) between ATN and FTN (15–17). These reports have mainly focused on non-inferiority and operational convenience.

We hypothesized that the benefits of ATNs may be more pronounced in larger nodules. To test this, we conducted a retrospective, observational, monocentric study comparing ATNs and FTNs in patients with cytologically benign nodules ≥20 mL.

## 2 Methods

### 2.1 Study design

This observational, retrospective, single-center study aimed to evaluate the different outcomes between the use of ATNs and conventional FTNs, in the treatment of large (≥20 mL) symptomatic benign thyroid nodules.

We enrolled, all patients consecutively treated with ultrasound-guided RFA using an ATN at the Division of Endocrinology, Diabetology and Metabolic Diseases of the “Ordine Mauriziano” Hospital in Turin, between January 2018 and December 2023, were enrolled, provided they met the predefined inclusion and exclusion criteria. These patients were hereafter referred to as the “ATN group”. They were compared to a control group, hereafter referred to as the “FTN group”, consisting of patients treated during a comparable time period using an FTN, and who met the same criteria.

### 2.2 Inclusion and exclusion criteria

#### 2.2.1 Inclusion criteria

- Minimum age of 18 years;
- A minimum baseline volume of the treated nodule of at least 20 mL;
- Treatment of solitary thyroid nodules or dominant nodules within a multinodular goiter, whether autonomously functioning (AFTN) or non-functioning;
- Indication for treatment based on compressive and/or cosmetic symptoms related to thyroid disease, in the case of non-functioning nodules;
- Indication for treatment of hyperthyroidism due to nodular disease, in the case of AFTN;
- Cytological exclusion of malignant thyroid disease, according to the 2014 SIAPEC classification, with particular reference to categories “indeterminate nodule at high risk (TIR3B)”, “suspicious for malignancy (TIR4)” and “malignant (TIR5)”;
- Exclusion of medullary thyroid carcinoma (MTC) through a one-time serum calcitonin measurement;
- Provision of informed consent for treatment with RFA and for the collection of clinical data for observational research purposes.

#### 2.2.2 Exclusion criteria

- Lack of ultrasound follow-up including the 12-month evaluation after treatment;
- Treatment of nodules with clearly suspicious initial ultrasound features (EU-TIRADS 4–5), such as irregular margins, presence of micro- or macrocalcifications, or marked hypoechogenicity, even in the presence of subsequent benign cytology;
- Clinical indications different from those listed under “Inclusion Criteria,” such as growing nodules without compressive symptoms, or symptomatic multinodular goiters without a clearly dominant nodule, treated for purely palliative purposes in elderly patients for whom surgery would have otherwise been the first-line option;
- Pregnant patients.

Thyroid functional status, as well as ongoing treatment with antithyroid drugs or levothyroxine replacement therapy, were not considered as indications for treatment and therefore did not represent exclusion criteria; such patients were included in the study.

Patients who had previously undergone thermal ablation for the same nodular disease—either during the study period or before—were not excluded, regardless of the technique used (RFA, laser ablation, microwave ablation). However, this information was systematically collected and considered as a potential confounding factor in the analysis.

### 2.3 Procedure

#### 2.3.1 Pre-treatment assessment

All patients underwent a thorough evaluation prior to being scheduled for RFA treatment, including:

- Clinical evaluation: Each patient underwent a physical examination to assess whether the target nodule was palpable, documenting its location, consistency, mobility, and/or visible appearance when applicable. During the same evaluation, both the Compressive Score and Cosmetic Score were assessed.
  - The *Compressive Score* is a subjective, patient-reported scale ranging from 0 to 10, reflecting the impact of compressive symptoms (e.g., dysphonia, dyspnea, dysphagia, and sensation of neck tightness or a foreign body) on quality of life, where 0 indicates no symptoms and 10 indicates maximum tolerable discomfort.
  - The *Cosmetic Score* is an objective, physician-assigned scale ranging from 1 to 4 based on the nodule’s visibility and palpability during physical examination: 1 = no palpable mass; 2 = palpable but not visible nodule with minimal cosmetic concern; 3 = nodule visible only during swallowing; 4 = nodule clearly visible at rest.
- Biochemical evaluation, including: complete blood count, coagulation profile, liver function tests, renal function, thyroid function tests, serum calcitonin (if never previously measured), and anti-thyroglobulin antibodies (AbTg).
- Thorough ultrasound examination, including detailed characterization of the nodule;
- Cytological evaluation: Fine-needle aspiration biopsy under ultrasound guidance was performed twice for nodules with non-clearly benign ultrasound features. A single biopsy was considered sufficient to exclude malignancy in cases of nodules with benign ultrasound appearance or autonomously AFTN, confirmed on scintigraphy, with suppressed TSH and clinical hyperthyroidism.
- Baseline electrocardiogram and the assessment of vocal cord function via laryngoscopy examination;

#### 2.3.2 Treatment session

RFA procedures were performed in day-hospital setting. Each procedure was conducted with the patient in the supine position and the neck hyperextended, according to a standardized protocol beginning with a repeated ultrasound assessment to confirm pre-admission findings and to plan the treatment strategy.

In addition to confirming nodule volume and dimensions, a detailed evaluation of vascularization was performed using color Doppler ultrasound, aiming to identify vascular structures to be ablated in order to minimize the risk of nodule regrowth over time. In nodules appearing hypovascular on Doppler imaging, contrast-enhanced ultrasound (CEUS) with 2.5 mL of sulfur hexafluoride microbubbles (Sonovue^©^), was implemented to better visualize microvascular architecture,

After the imaging assessment, local anesthesia was administered under ultrasound guidance: 10 mL of a solution composed of 8 mL of 2% mepivacaine and 2 mL of 1.4% sodium bicarbonate was injected into the virtual space between the thyroid gland and the strap muscles.

A trans-isthmic, medio-lateral approach was used for needle insertion.

In the *ATN group*, treatment was performed using a VIVA RF Electrode (STARmed Co., Ltd.), 18G in diameter, 7 cm in length, and an adjustable active tip ranging from 5 to 30 mm. The choice of active tip length was based on the size, composition, location of the nodule, and the power required.

In the FTN group, a Star RF Electrode - Fixed (STARmed Co., Ltd.) with a fixed 10 mm active tip, 18G diameter, and 7 cm shaft length was used.

Both electrodes were connected to a VIVA RF Generator (STARmed Co., Ltd.). Power settings (W) were selected according to the active tip length and the internal and volumetric characteristics of the nodule; ablation was usually initiated at 40–60 W and adjusted thereafter.

A moving-shot technique was adopted for all procedures. When peripheral or feeding arterioles were identified within the nodule, they were targeted and ablated first.

In nodules considered at increased risk of complications due to their size or proximity to critical structures, the hydrodissection technique was used with local anesthetic to create a protective barrier.

At the end of the procedure, if CEUS had been performed during the pre-treatment evaluation, a second 2.5 mL dose of contrast agent was administered to assess the extent of ablation and detect any viable residual areas requiring immediate completion.

All ultrasound evaluations, including image guidance during the procedure, were performed using a MyLab XPro80 ultrasound system (Esaote S.p.A.) equipped with a 4–15 MHz linear probe.

Upon completion of the procedure, based on the treated nodule’s size, the duration of the intervention, and the patient’s reported symptoms, a 4 mg intravenous dose of betamethasone was administered. Each patient was monitored for at least 2 hours before being discharged with instructions regarding the recognition of potential complications, supportive therapy (a 3-day antibiotic prophylaxis, usually azithromycin 500 mg, and pain relief with paracetamol 1 g as needed), and scheduled follow-up appointments.

All patients were subsequently re-evaluated at 1, 6, and 12 months.

### 2.4 Collected data

At baseline, all data from pre-procedural evaluations (anthropometric, clinical, biochemical, and ultrasound parameters) were collected, along with all procedural variables, including: average power (W), procedure duration, use of contrast agent, total energy delivered (kJ), delivered energy (deposited energy per unit of baseline volume) (kJ/mL), and energy/time ratio (deposited energy per unit of time) (kJ/min).

At each follow-up visit (1, 6, and 12 months), the following were assessed: Volume reduction ratio (VRR), calculated as: $VRR=\frac{\left(Volume_{baseline}-Volume_{at\;the\;time}\right)}{Volume_{baseline}}\;\times 100;$ Compressive and Cosmetic Scores.

Additionally, at the 6-month visit, thyroid function tests (TSH, fT4, fT3) were re-evaluated, except for AFTN patients, for whom thyroid function was assessed earlier, at 3 months post-treatment.

### 2.5 Statistical analysis

Statistical analyses were performed using R software (version 4.4.2; R Core Team, 2022), Continuous variables were summarized using the median and interquartile range (IQR). Categorical variables were reported as absolute numbers and percentages. Normality of distribution for continuous variables was assessed using the Shapiro–Wilk test. At baseline, categorical variables were compared using the chi-square test; when expected frequencies were<5, Fisher’s exact test was applied. For continuous variables, comparisons were performed using the Student’s t-test if normally distributed, or the Mann–Whitney U test otherwise. To compare two continuous variables, the Spearman correlation test was used. Group comparisons were first conducted in univariate analysis by calculating the area under the curve (AUC) for each patient and comparing AUC distributions between groups using the Mann–Whitney U test. Further evaluation was conducted using multivariate generalized linear mixed models, which allowed adjustment for both random effects (interindividual variability) and fixed effects (e.g., treatment group and identified confounders), as well as interaction terms over time for longitudinal analysis. Missing data were managed using the last observation carried forward (LOCF) method. The threshold for statistical significance (α) was set at 0.05.

## 3 Results

### 3.1 Population

A total of 75 patients were enrolled: 38 in the FTN group and 37 in the ATN group. The characteristics of the study population are summarized in **Table 1**. At the time of the procedure, the median age was 57.61 years (48.85 – 67.10). The cohort included 52 (69.33%) women, with a female-to-male ratio of approximately 2.26:1. Multinodular disease was present in 49 (65.33%) cases. The treated nodules had a median volume of 36.10 mL (31.63 – 51.25) and a median maximum diameter of 52.00 mm (47.00 – 59.00). In the majority of cases, 72 (96%), the indication for treatment was symptomatic benign nodular disease. The remaining 3 (4%) patients had AFTN. Most nodules had a cytological diagnosis of benign lesion (TIR2) in 72 (96%) patients. The remaining 3 (4%) had a low-risk indeterminate cytology (TIR3A). Among these, two were AFTNs without suspicious ultrasound features, while the third occurred in a patient with compressive symptoms who was not eligible for surgery and showed no suspicious mutations or rearrangements on cytological molecular analysis. The median baseline compressive score reported by patients was 3 (2 – 5). The most represented Cosmetic score class was the III one (43 patients, 57.34%).

**Table 1:** Summary of baseline features of patients and treated nodules both for the entire cohort and stratified by treatment group.

Characteristics	Population (n= 75)	FTN (n = 38)	ATN (n = 37)	*p.*
Age (years)	57.61 (48.85 – 67.10)	55.63 (46.47 – 63.58)	60.48 (50.10 – 70.12)	*0.066*
Gender (Females)	52 (69.33%)	26 (68.42%)	26 (70.27%)	*0.939*
Multinodular disease	49 (65.33%)	23 (60.53%)	26 (70.27%)	*0.520*
Baseline volume (mL)	36.10 (31.63 – 51.25)	37.80 (32.40 – 52.70)	34.20 (29.18 – 43.65)	*0.131*
Larger diameter (mm)	52.00 (47.00 – 59.00)	55.50 (49.00 – 59.00)	50.00 (47.00 – 58.50)	*0.205*
Cytology				*0.115*
*TIR2*	72 (96%)	38 (100%)	34 (91.89%)	
*TIR3A*	3 (4%)	0 (0%)	3 (8.11%)	
Indication				*0.615*
*Symptoms*	72 (96%)	37 (97.37%)	35 (94.60%)	
*AFTN*	3 (4%)	1 (2.63%)	2 (5.40%)	
Compressive score(pt)	3 (2 - 5)	3 (1 – 5)	4 (2 – 6)	*0.130*
Cosmetic score				*0.688*
*I*	0 (0)	0 (0)	0 (0)	
*II*	19 (25.33%)	8 (21.05%)	11 (29.73%)	
*III*	43 (57.34%)	23 (60.53%)	20 (54.05%)	
*IV*	13 (17.33%)	7 (10.53%)	6 (16.22%)	
Baseline TSH (mUI/L)	1.10 (0.58 – 1.41)	0.84 (0.58 – 1.39)	1.16 (0.65 – 1.46)	*0.185*
Baseline AbTG titer (kUI/L)	1.63 (0.91 – 4.57)	1.77 (0.84 – 5.08)	1.59 (0.93 – 4.12)	*0.787*
Baseline AbTG positivity (%)	19 (25.33%)	9 (18.42%)	10 (27.03%)	*0.946*

No statistically significant differences were observed between the two groups. The Mann–Whitney test was used for continuous variables, the chi-square test for categorical variables, and Fisher’s exact test for categorical variables with fewer than five observations. The level of statistical significance (α) was set at 0.05. FTN, fixed-tip needle; ATN, adjustable-tip needle; AFTN, autonomous functioning thyroid nodule; TSH, thyrotropin; AbTG, anti-Thyroglobulin antibodies.

No statistically significant differences were found between the FTN and ATN groups at baseline (**Table 1**).

### 3.2 Procedural features

**Table 2** summarizes the features of the ablative treatment. Most patients (68, 90.67%) underwent their first procedure. In 4 patients (5.33%), the treatment followed a prior Percutaneous Ethanol Injection (PEI) targeting cystic components. The procedure had a median duration of 14.87 (12.64 – 19.89) minutes, with a median power output of 70 (60.00 – 88.12) W, resulting in a total energy delivery of 51.38 (44.11 – 67.93) kJ, and a corresponding delivered energy of 1.45 (1.04 – 1.91) kJ/mL.

**Table 2:** Summary of baseline features related to procedures, both for the entire cohort and stratified by treatment group.

Characteristics	Population (n= 75)	FTN (n = 38)	ATN (n = 37)	*p.*
Number of sessions (1 *vs >*1)	68 (90.67%)	38 (100%)	30 (83.78%)	*0.06*
Previous PEI	4 (5.33%)	2 (5.26%)	2 (5.41%)	*1.000*
Session duration (min)	14.87 (12.64 – 19.89)	15.59 (13.55 – 20.02)	14.25 (12.35 – 18.59)	*0.231*
Total Energy (kJ)	51.38 (44.11 – 67.93)	44.71 (34.06 – 50.79)	64.39 (53.41 – 92.13)	***< 0.001***
Delivered energy (kJ/mL)	1.45 (1.04 – 1.91)	1.15 (8.08 – 1.38)	1.92 (1.74 – 2.09)	***< 0.001***
Energy per minute (kJ/min)	3.38 (2.58 – 4.39)	2.59 (2.39 – 2.73)	4.41 (4.12 – 5.46)	***< 0.001***
Mean power (W)	70.00 (60.00 – 88.13)	60.00 (55.00 – 60.00)	90.00 (80.00 – 100.00)	***< 0.001***
CEUS employment	19 (25.33%)	7 (18.4%)	12 (32.43%)	*0.259*
Early subjective success	49 (65.33%)	26 (68.42%)	23 (62.16%)	*0.744*
Minor complications	6 (8.00%)	2 (5.26%)	4 (10.81%)	*0.430*

Only delivered energy, energy per minute ratio, and mean generated power were significantly higher in the ATN group. The Mann–Whitney test was used for continuous variables, the chi-square test for categorical variables, and Fisher’s exact test for categorical variables with fewer than five observations. The level of statistical significance (α) was set at 0.05. FTN, fixed-tip needle; ATN, adjustable-tip needle; PEI, percutaneous ethanol injection; CEUS, contrast-enhanced ultrasound.Bold-format of values in those and all tables stands for "p-values <0.05" and consequently, statistically significant features.

CEUS was used in 19 patients (25.33%). Six patients (8%) experienced self-limiting adverse effects or minor complications during or immediately after the procedure, all resolving within one month or after short oral courses of corticosteroids or antibiotics.

In ATN group, median exposure of the active tip resulted 15.00 (15.00 – 20.00) mm.

The only statistically significant differences between the ATN and FTN groups (**Table 2**) were observed for: power output (64.39 (53.41 – 92.13) W vs. 44.71 (34.06 – 50.79) W, p< 0.001), delivered energy (1.92 (1.74 – 2.09) kJ/mL vs. 1.15 (1.08 – 1.38) kJ/mL, p< 0.001), and energy per minute (4.41 (4.12 – 5.46) kJ/min vs. 2.59 (2.39 – 2.73) kJ/min, p< 0.001), all higher in the ATN group. No significant differences were found for other variables, including procedure duration, operator’s immediate estimation of treatment success, or complication rates.

### 3.3 Volume reduction and clinical outcomes

**Table 3** summarizes dimensional and clinical outcomes at 12 months. At that time, the overall cohort reached a median VRR of 58.52% (46.00–70.14). All patients reported complete resolution of compressive symptoms (CpS 0 (0–0)), and only 2 patients (2.67%) still presented with a clinically detectable cervical swelling (CmS 4/4).

**Table 3:** Summary of dimensional and clinical outcomes at 12-month follow-up, both for the entire cohort and stratified by treatment group.

Characteristics	Population (n= 75)	FTN (n = 38)	ATN (n = 37)	*p.*
VRR (%)	58.52 (46.00 – 70.14)	52.44 (33.47 – 60.52)	60.48 (56.32 – 72.55)	***0.004***
Volume (mL)	16.00 (11.08 – 28.73)	18.40 (12.80 – 32.40)	14.10 (8.35 – 18.23)	***0.012***
Larger diameter (mm)	39.00 (33.00 – 49.00)	41.00 (36.00 – 52.00)	37.00 (32.00 – 43.00)	***0.014***
Compressive score (pt)	0.00 (0.00 – 0.00)	0.00 (0.00 – 0.00)	0.00 (0.00 – 0.75)	*0.555*
Cosmetic score (class)				*0.905*
*I*	5 (6.67%)	2 (5.26%)	3 (8.10%)	
*II*	26 (34.67%)	15 (39.47%)	11 (29.73%)	
*III*	7 (9.33%)	4 (10.53%)	3 (8.10%)	
*IV*	2 (2.67%)	1 (2.63%)	1 (2.70%)	

The ATN group showed significantly lower values for nodule volume, larger diameter, and VRR at 12 months. The Mann–Whitney test was used for continuous variables, the chi-square test for categorical variables, and Fisher’s exact test for categorical variables with fewer than five observations. The level of statistical significance (α) was set at 0.05. FTN, fixed-tip needle; ATN, adjustable-tip needle; VRR, volume-reduction ratio.Bold-format of values in those and all tables stands for "p-values <0.05" and consequently, statistically significant features.

At 12 months, patients in the ATN group showed significantly lower volumes (14.10 (8.35–18.23) mL vs 18.40 (12.80–32.40) mL, p = 0.012), maximum diameters (37.00 (32.00–43.00) mm vs 41.00 (36.00–52.00) mm, p = 0.014), and VRRs (60.48 (56.32–72.55) % vs 52.44 (33.47–60.52) %, p = 0.004). No significant differences were observed in clinical scores (CpS: p = 0.553, CmS: 0.905).

**Figure 1:**
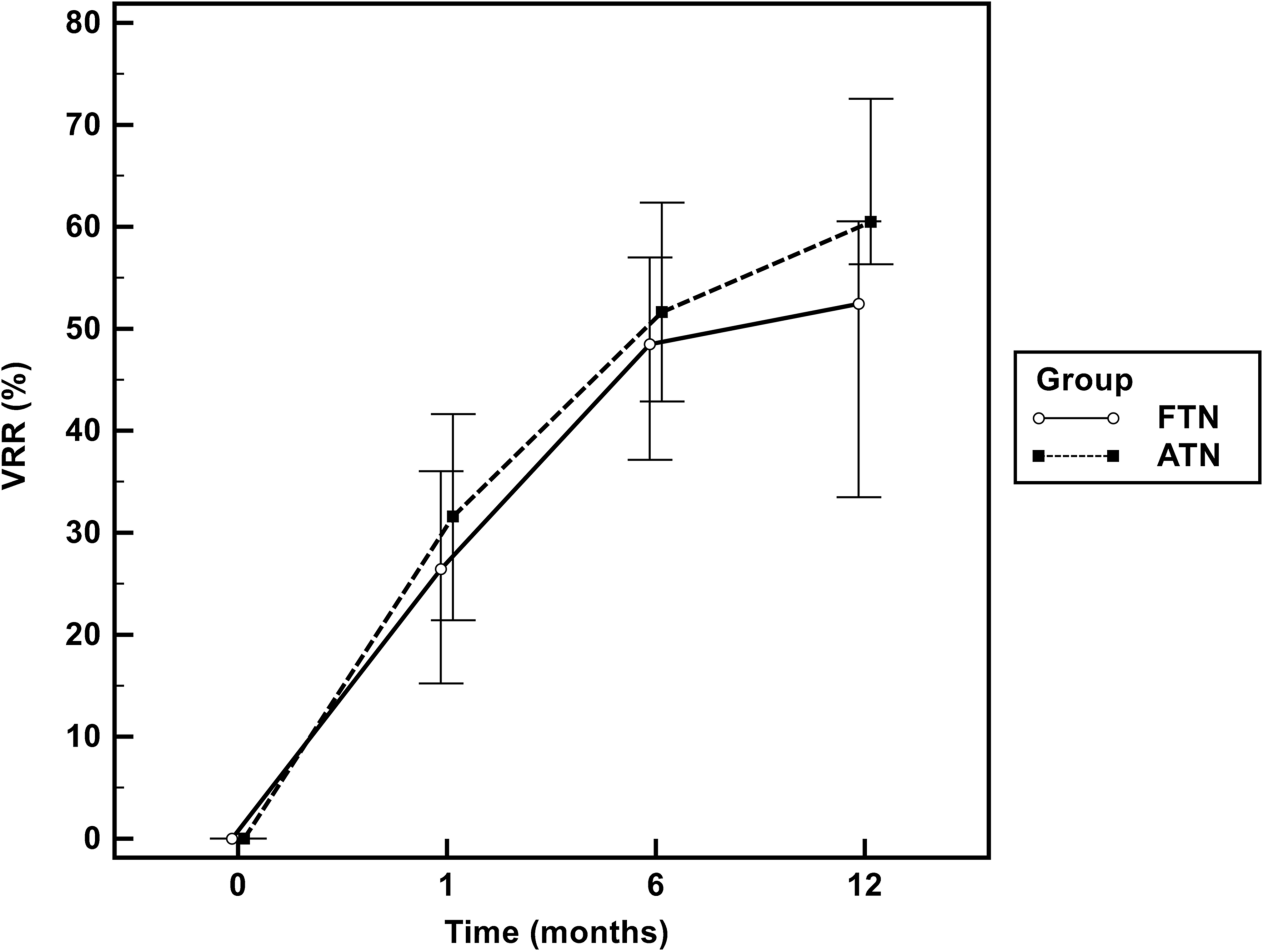
VRR trend during follow-up, in the two study groups. FTN, fixed-tip needle; ATN, adjustable-tip needle.

In the assessment of longitudinal trends in dimensional and clinical outcomes, based on comparisons of the median AUCs of each variable between groups, the ATN group showed a consistently higher VRR throughout follow-up (AUC_ATN_: 551.44 (490.88–600.50) vs AUC_FTN_: 478.83 (430.23–535.14), p = 0.019) (**Figure 1**) and a consistently lower volume (AUC_ATN_: 219.45 (181.97–251.38) vs AUC_FTN_: 279.43 (220.61–378.65), p = 0.040). Maximum diameter also tended to be lower in the ATN group, although the difference only approached statistical significance (AUC_ATN_: 490.98 (446.73–534.09) vs AUC_FTN_: 536.50 (496.12–574.07), p = 0.061).

### 3.4 Features predicting reduction

At univariate analyses, the 12-month VRR in the overall population was reduced by higher baseline volume (ρ = –0.40, p< 0.001), baseline maximum diameter (ρ = –0.25, p = 0.034), and treatment duration (ρ = –0.16, p = 0.020), while it resulted greater in ATN group (p = 0.004), higher median power (ρ = 0.25, p = 0.029), energy/time ratio (ρ = 0.1380, p = 0.020) and delivered energy (ρ = 0.43, p< 0.001).

No other significant associations were observed with the remaining variables analyzed, including cytological outcome, treatment indication, retreatment, use of CEUS, initial estimation, age, and thyroid function.

Variables showing significant inter-correlation and therefore excluded from subsequent multivariate analyses due to the risk of collinearity were: treatment duration (correlated with baseline volume), mean power (associated with the study group), delivered energy (associated with the study group and baseline volume), baseline maximum diameter (correlated with baseline volume), and energy per minute (associated with the study group).

#### 3.4.1 Multivariate analysis

**Table 4** shows the output of the generalized linear mixed model used to assess the effect of significant, non-collinear variables derived from the previous analyses, as well as their interactions over the follow-up period, in predicting VRR, while accounting for the expected interindividual variability of the response variable.

**Table 4:** Report of the generalized linear mixed model with VRR as the dependent variable and study group, follow-up time, baseline volume, and their interactions as independent variables.

Generalized linear mixed model fit by maximum likelihood (Laplace Approximation)				
Random effects:				
Groups	Name	Variance	Std.Dev.	
ID	(Intercept)	17478.3	132.21	
Residuals		202.7	14.24	
Fixed effects:				
Variable	Estimate	Std.Error	t-value	p-value
GROUP[ATN]	24.141	27.376	0.882	0.37787
**TIME**	**33.969**	0.2468	13.764	**< 0.001**
Basal_Volume	-43.311	38.422	-1.127	0.25964
**GROUP: TIME**	**11.169**	0.3491	3.199	**0.00138**
GROUP: Basal_volume	27.972	41.187	0.679	0.49704
**TIME: Basal_volume**	**-11.336**	0.4842	-2.341	**0.01922**
GROUP: TIME: Basal_volume	0.7020	0.5195	1.351	0.17659

Basal volume and the employment of ATNs proved a mutually independent time-dependent effect on VRR. Family: gaussian (identity). Formula: VRR ~ Group * Time * Basal_volume + (1 | ID). Number of observations: 285, groups: ID, 75.Bold-format of values in those and all tables stands for "p-values <0.05" and consequently, statistically significant features.

The following effects were found to be significant:

- Follow-up time, as an independent variable, with a positive coefficient (β = 3.397, p< 0.001);
- Interaction between the use of ATN and follow-up time, with a positive coefficient (β = 1.117, p = 0.001);
- Interaction between baseline volume and follow-up time, with a negative coefficient (β = –1.134, p = 0.019).

In summary, the use of ATN was associated with a greater increase in VRR over time, compared with FTN, irrespective of baseline volume, as was the progression of follow-up time. Conversely, larger baseline volumes were associated with a reduced treatment efficacy over time.

No independent effects of the variables, nor any volume-dependent effect of ATN, were observed.

### 3.5 Success rate: VRR ≥ 50%

The use of ATN was found to be associated with a higher success rate at 12 months, defined as VRR ≥ 50% (OR 4.18 (95% CI 1.42–12.35), p = 0.015).

This finding was confirmed by the generalized logistic regression model (**Table 5**), in which the use of ATN remained significantly associated with a higher probability of success (VRR ≥ 50%) at 12 months, independently of baseline volume (β = 1.79, p = 0.005). Conversely, baseline volume was found to predict a lower success rate at 12 months, independently of the treatment group (β = –0.03, p = 0.008).

**Table 5:** Report of the generalized linear mixed model with VRR as the dependent variable and study group, follow-up time, baseline volume, and their interactions as independent variables.

Generalized logistic model					
Coefficients:	Estimate	Std.Error	Odds Ratio (CI)	z-value	p-value
**GROUP[ATN]**	1.46953	0.65543	4.35 (1.30 - 18.02)	2.242	**0.02496**
**Basal_volume**	-0.03457	0.01256	0.97 (0.94 - 0.99)	-2.752	**0.00592**

Basal volume and ATNs proved their independent effect on success (VRR ≥ 50%) at 12 months.Family: binomial (logit). Formula: SUCC50_12 ~ GROUP + Basal_volume. Dispersion parameter for binomial family taken to be 1. Null deviance: 92.461 on 74 degrees of freedom. Residual deviance: 74.741 on 72 degrees of freedom.Bold-format of values in those and all tables stands for "p-values <0.05" and consequently, statistically significant features.

**Figure 2:**
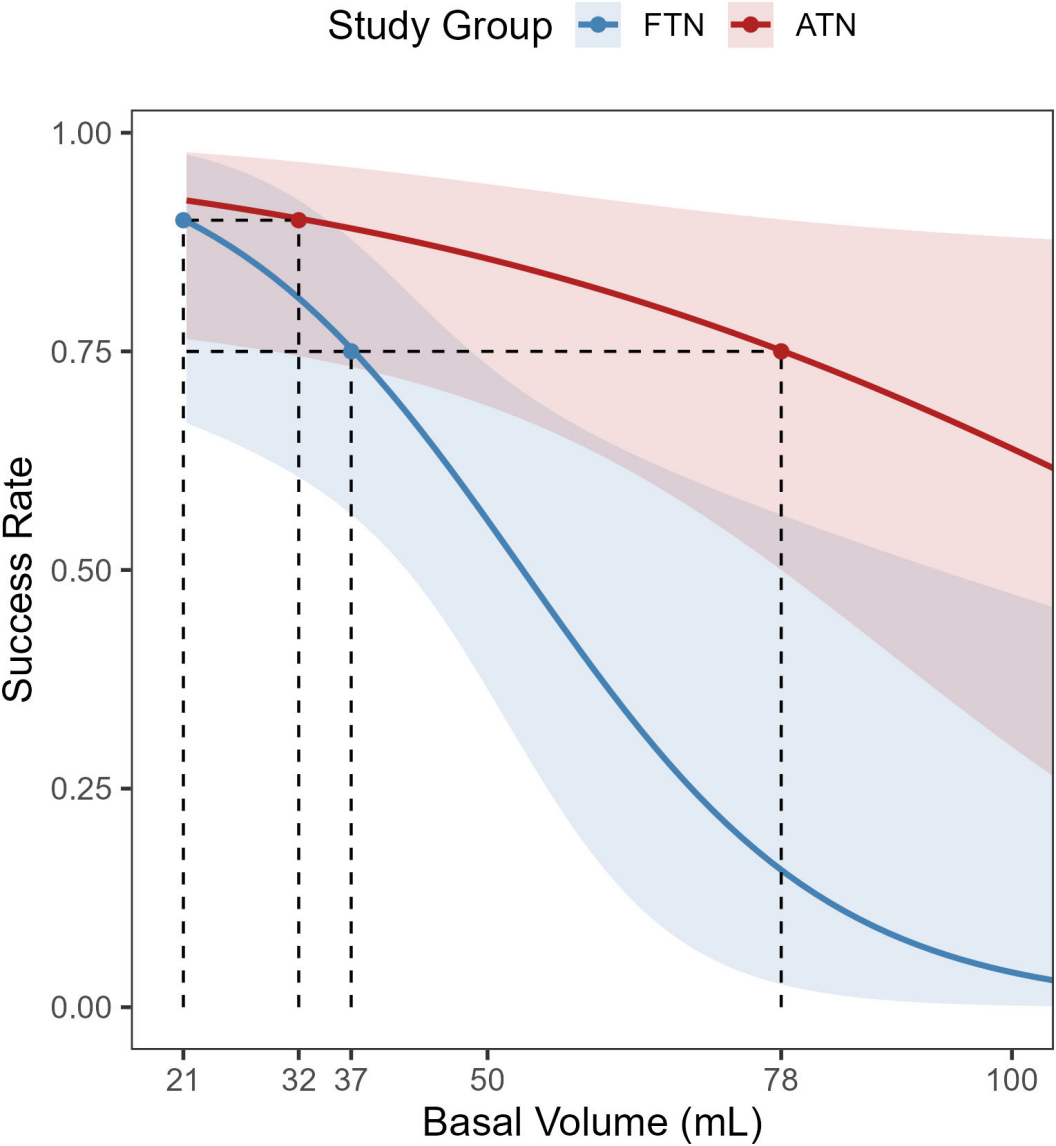
Probability of success, defined as achieving VRR ≥ 50%, according to baseline volume in the two study groups, as predicted by the logistic regression model. The volumetric threshold at which the probability of success fell below 90% was 32 mL for ATN and 21 mL for FTN. When considering success probability below 75%, the cut-off increased to 37 mL for FTN and to 78 mL for ATN.

We applied a logistic regression model to estimate the probability of success according to baseline volume in the two study groups. The volumetric threshold at which the probability of success fell below 90% was 32 mL for ATN and 21 mL for FTN. When considering success probability below 75%, the cut-off increased to 37 mL for FTN and to 78 mL for ATN. The corresponding probability curves are shown in **Figure 2**.

## 4 Discussion

In this retrospective, single-center observational study, we aimed to compare the efficacy of RFA using an ATN versus the more conventional FTN in the treatment of benign thyroid nodules.

RFA confirmed its overall efficacy, as both groups demonstrated, at 12 months of follow-up, a median VRR consistent with data reported in the literature (50–80%) (18). Specifically, the median VRR observed in our ATN group (60.48%) was comparable to that reported in the only other study assessing 12-month outcomes with variable-tip electrodes (68.3%) (15).Conversely, it was lower than that reported by Shin et al. (16), who observed a median reduction of 83.3% (70.3–98.1%). However, that study did not assess VRR at predefined time points but rather reported each patient’s best outcome during follow-up, later categorized as “more than 2 years” or “less than 2 years.” Therefore, their results reflect a somewhat longer and not directly comparable observation period. Moreover, a substantial proportion of nodules in that cohort (n = 24) required multiple treatment sessions.

Another relevant difference between the two studies concerns the baseline nodule volume, which in Shin et al. was significantly smaller (9.9 (5.1–19.7) mL) than in our ATN group (34.20 (29.18–43.65) mL) (16). It is well established that baseline nodule volume is one of the main predictors of poorer outcomes (19), as larger nodules make it more difficult to achieve complete treatment and to ablate peripheral regions of the lesion. Indeed, a sub-analysis of the same study focusing on nodules >10 mL—an inclusion threshold still lower than that of our population (>20 mL)—showed lower results for a similar follow-up duration (“less than 2 years”), with a mean VRR of 75.4 ± 17%.

The central role of baseline volume also emerged from our analysis, where larger initial volumes showed a strong negative correlation with 12-month VRR and significantly lower probabilities of treatment success. Multivariate analysis further confirmed a time-dependent relationship, whereby greater baseline volumes were associated with slower VRR reduction over time and delayed achievement of success (VRR > 50%).

In our experience, the use of the ATN was associated with a consistently greater VRR throughout follow-up at both 6 and 12 months, as well as with smaller final nodule volumes. This was evident both in longitudinal analysis (with a significantly higher AUC) and in point-by-point comparisons at each follow-up. At 6 months after treatment, the median VRR in the ATN group was already higher than in the FTN group (51.62% vs. 48.49%), and this difference persisted at 12 months, when patients treated with ATN showed significantly lower nodule volumes (14.10 mL vs. 18.40 mL), maximum diameters (37 mm vs. 41 mm), and higher VRR values (60.48% vs. 52.44%) compared with the FTN group. In our selected population, these findings were evident both as a time-dependent effect—suggesting a faster achievement of therapeutic response—and as an effect independent of potential confounders, such as minor interindividual variability or differences in baseline nodule volume.

The advantage of ATN use was further confirmed when evaluating treatment success, defined as a ≥50% reduction in nodule volume. The ATN group showed a higher probability of success independently of baseline volume.

To date, our study represents the first reported experience demonstrating an actual superiority of ATNs. Indeed, the two previous comparative studies available in the literature found no significant differences in clinical outcomes between ATN and FTN, either at 6 months (17) or with a roughly “greater than 2 years” follow-up (16).

These findings may support our working hypothesis that the real advantage of this technology lies in its improved efficacy in treating large nodules (>20 mL), a clinical setting in which several studies have documented the relative failure of fixed-tip electrodes (FTN) (8, 10, 20). In our population, the probabilistic relationship between baseline volume and treatment success allowed us to infer a volumetric threshold beyond which the probability of achieving success markedly decreased. This threshold was approximately 35 mL for FTN—consistent with the aforementioned studied (20)—and substantially higher, at 80 mL, for ATN.

When comparing baseline and procedural characteristics between the two groups to identify potential explanations for the observed efficacy differences, the only variables found to be significantly higher in the ATN group were energy-related parameters: total energy (64.39 (53.41–92.13) kJ vs. 44.71 (34.06–50.79) kJ), delivered energy per mL (1.92 (1.74–2.09) kJ/mL vs. 1.15 (0.80–1.38) kJ/mL), energy per minute (4.41 (4.12–5.46) kJ/min vs. 2.59 (2.39–2.73) kJ/min), and mean power (90.00 (80.00–100.00) W vs. 60.00 (55.00–60.00) W).

These findings are consistent with the hypothesis we proposed in a previous study conducted in a different population (17), although they contrast with the only other available report (16). Notably, both generated power (r = 0.25, p = 0.029), energy/time ratio (ρ = 0.1380, p = 0.020) and delivered energy (r = 0.43, p< 0.001) emerged as independent predictors of greater treatment efficacy, irrespective of electrode type, in line with previous evidence (21, 22).

From a causal perspective, the higher energy delivery observed in the ATN group should not be interpreted as a confounding factor, but rather as a mechanistic mediator of the treatment effect. In other words, the electrode type is not expected to exert a direct biological effect per se; instead, its clinical advantage plausibly derives from its technical capability to deliver higher and more homogeneous thermal energy over a larger ablation volume. Adjusting for delivered energy in multivariable models would therefore represent an overadjustment, as it would partially control for a variable lying on the causal pathway between electrode design and treatment efficacy, potentially attenuating the true effect of ATN.

Interestingly, the median duration of treatment did not differ between groups: 15.59 minutes for FTN and 14.25 minutes for ATN. This finding is only partially consistent with the literature. Similarly, Rossi et al. did not report significant differences in treatment duration, whereas Shin et al. observed a significant reduction in procedure time with ATN, particularly for larger nodules (17). Reported values, however, vary widely across studies for both FTN and ATN: 18.5 (14.28–21.70) min vs. 13.0 (10.08–16.15) min (16); 16 (11–17) min vs. 19 (15–25) min (17), and 22 (9–36) min (15). Therefore, treatment duration appears to depend more on operator-related factors (experience, technique, intent) and treated volume, rather than representing a consistent or easily comparable variable.

Overall, these results support the hypothesis that ATNs allow for a more homogeneous and spatially extended distribution of thermal energy across the ablation zone. This is reflected in higher energy delivery per minute and per unit volume, without a reduction in ablation time. Rather than indicating reduced procedural efficiency, this finding likely reflects a different pattern of thermal propagation, characterized by more gradual and uniform energy deposition. The longer active tip may reduce the need for repeated probe repositioning and improve coverage of larger treatment volumes, thereby optimizing tissue coagulation while limiting focal overheating phenomena such as carbonization, which can impair energy transmission.

Although higher energy delivery has been theoretically associated with an increased risk of complications (including recurrent laryngeal nerve injury and nodule rupture) (23), current evidence does not support a consistent dose–response relationship between total energy output and adverse events (24). Instead, procedural safety appears primarily dependent on technical factors such as operator experience, precise probe positioning, and strict adherence to anatomical safety margins, including the thyroid capsule and the so-called “danger triangle” (5, 7). In this context, individualized modulation of active-tip length and power output is likely more relevant than absolute energy delivery in determining safety outcomes.

In fact, ATN use was not associated with higher complication or side-effect rates. Overall, the incidence of adverse events was very low (8%), and all were minor and self-limiting, resolving without sequelae. This is consistent with previous reports (4) and further supports the safety of the procedure when performed by experienced operators. Importantly, no increase in complication rates was observed despite the higher energy delivery associated with ATN use.

Regarding clinical outcomes, there were no significant differences between the two groups in either compressive symptom scores or cosmetic scores. Treatment led to complete resolution of compressive symptoms in all patients. These findings confirm the positive impact of RFA on patient quality of life and are consistent with previous evidence by Kim et al. (7), who reported clinical improvement in 90–95% of treated patients. The strength of our study lies in being the first report in the literature to clearly identify the specific clinical setting in which the ATN demonstrates superiority over standard FTN—namely, in the treatment of larger nodules, particularly those exceeding 20 mL in volume. The main limitation of our study is the relatively short follow-up period (12 months), which ensured good comparability among all patients but did not allow for long-term evaluations, particularly regarding regrowth rates, the potential need for retreatment and the temporal evolution of volume reduction. In particular, it cannot be excluded that the differences observed between groups at 12 months may attenuate over time, as volume reduction in FTNs has been reported to progressively increase beyond the first year (25). In addition, due to the retrospective design, the ablation rate could not be systematically assessed, as this parameter was not consistently reported in earlier clinical records and could not be reliably reconstructed from archived imaging. This may represent a limitation, as the ablation rate provides additional information on procedural completeness and could further refine the comparison between groups, particularly in relation to treatment efficacy and the risk of residual viable tissue.

## 5 Conclusions

This retrospective, observational, single-center study demonstrates that, in the radiofrequency ablation of benign thyroid nodules with a baseline volume >20 mL, the use of an ATN is associated with a greater volume reduction within 12 months compared to FTN, as well as a higher likelihood of achieving the therapeutic success threshold of 50% VRR at this time point, suggesting a faster treatment response compared to the standard approach. To our knowledge, this is the first study to provide scientific evidence of this finding, complementing a few sporadic reports conducted on smaller nodules.

The observed efficacy may be attributed to the inclusion of only nodules >20 mL in our sample, which are classically considered more challenging, as standard FTN techniques often show limited effectiveness in this setting, whereas ATN appears to maintain the expected outcomes. Specifically, our data suggest that this advantage is related to the ATN’s ability to modulate the active-tip length, enabling more efficient energy delivery over a larger surface area. This allows for the treatment of larger volumes, optimizing heat distribution while concurrently minimizing the risk of local carbonization.

## Data Availability

The raw data supporting the conclusions of this article will be made available by the authors, without undue reservation.
